# A diagnostic and epidemiologic investigation of acute febrile illness (AFI) in Kilombero, Tanzania

**DOI:** 10.1371/journal.pone.0189712

**Published:** 2017-12-29

**Authors:** Christine Hercik, Leonard Cosmas, Ondari D. Mogeni, Newton Wamola, Wanze Kohi, Victor Omballa, Melvin Ochieng, Shirley Lidechi, Juma Bonventure, Caroline Ochieng, Clayton Onyango, Barry S. Fields, Sayoki Mfinanga, Joel M. Montgomery

**Affiliations:** 1 Georgetown University, Washington, DC, United States of America; 2 Disease Detection Branch, Division of Global Health Protection, US Centers for Disease Control and Prevention (CDC), CDC-Kenya, Nairobi, Kenya; 3 Kenya Medical Research Institute-Centre for Global Health Research (KEMRI-CGHR), Nairobi, Kenya; 4 National Institute of Medical Research (NIMR) Muhimbili Research Centre, Dar es Salaam, Tanzania; 5 Division of Global Health Protection, Center for Global Health, CDC, Atlanta, GA, United States of America; Kliniken der Stadt Köln gGmbH, GERMANY

## Abstract

**Introduction:**

In low-resource settings, empiric case management of febrile illness is routine as a result of limited access to laboratory diagnostics. The use of comprehensive fever syndromic surveillance, with enhanced clinical microbiology, advanced diagnostics and more robust epidemiologic investigation, could enable healthcare providers to offer a differential diagnosis of fever syndrome and more appropriate care and treatment.

**Methods:**

We conducted a year-long exploratory study of fever syndrome among patients ≥ 1 year if age, presenting to clinical settings with an axillary temperature of ≥37.5°C and symptomatic onset of ≤5 days. Blood and naso-pharyngeal/oral-pharyngeal (NP/OP) specimens were collected and analyzed, respectively, using AFI and respiratory TaqMan Array Cards (TAC) for multi-pathogen detection of 57 potential causative agents. Furthermore, we examined numerous epidemiologic correlates of febrile illness, and conducted demographic, clinical, and behavioral domain-specific multivariate regression to statistically establish associations with agent detection.

**Results:**

From 15 September 2014–13 September 2015, 1007 febrile patients were enrolled, and 997 contributed an epidemiologic survey, including: 14% (n = 139) 1<5yrs, 19% (n = 186) 5-14yrs, and 67% (n = 672) ≥15yrs. AFI TAC and respiratory TAC were performed on 842 whole blood specimens and 385 NP/OP specimens, respectively. Of the 57 agents surveyed, *Plasmodium* was the most common agent detected. AFI TAC detected nucleic acid for one or more of seven microbial agents in 49% of AFI blood samples, including: *Plasmodium* (47%), *Leptospira* (3%), *Bartonella* (1%), *Salmonella enterica* (1%), *Coxiella burnetii* (1%), *Rickettsia* (1%), and West Nile virus (1%). Respiratory TAC detected nucleic acid for 24 different microbial agents, including 12 viruses and 12 bacteria. The most common agents detected among our surveyed population were: *Haemophilus influenzae* (67%), *Streptococcus pneumoniae* (55%), *Moraxella catarrhalis* (39%), *Staphylococcus aureus* (37%), *Pseudomonas aeruginosa* (36%), Human Rhinovirus (25%), influenza A (24%), *Klebsiella pneumoniae* (14%), Enterovirus (15%) and group A *Streptococcus* (12%). Our epidemiologic investigation demonstrated both age and symptomatic presentation to be associated with a number of detected agents, including, but not limited to, influenza A and *Plasmodium*. Linear regression of fully-adjusted mean cycle threshold (C_t_) values for *Plasmodium* also identified statistically significant lower mean C_t_ values for older children (20.8), patients presenting with severe fever (21.1) and headache (21.5), as well as patients admitted for in-patient care and treatment (22.4).

**Conclusions:**

This study is the first to employ two syndromic TaqMan Array Cards for the simultaneous survey of 57 different organisms to better characterize the type and prevalence of detected agents among febrile patients. Additionally, we provide an analysis of the association between adjusted mean C_t_ values for *Plasmodium* and key clinical and demographic variables, which may further inform clinical decision-making based upon intensity of infection, as observed across endemic settings of sub-Saharan Africa.

## Introduction

With gains in vector control across sub-Saharan Africa and a significant decrease in overall malaria incidence rates, a better understanding of the prevalence of both malarial and non-malarial agents potentiating fever syndrome is needed for several reasons including efforts to more rapidly detect and control diseases at their source to ensure global health security [1–5]. In low-resource settings, limited access to clinical laboratory diagnostics requires empiric case management of febrile illness. The use of comprehensive fever syndromic surveillance, with enhanced clinical microbiology, advanced diagnostics and more robust epidemiologic investigation, could enable healthcare providers to offer a differential diagnosis of fever syndrome and more appropriate care and treatment [6]. In order to inform clinical management and optimize treatment regimens for febrile patients, information on common causes and associations of febrile illness, particularly in areas endemic to malaria, is critically required.

While previous studies have adopted a syndromic approach to estimate global burden of disease due to diarrhea and pneumonia, such an approach has not been taken to evaluate fever in the absence of other characterizing features (e.g., gastroenteritis, skin or soft tissue infections, etc.) [7–8]. Pathogen-specific approaches have been utilized to evaluate illness due to certain febrile diseases, predominately malaria, typhoid fever and arboviral infections, such as dengue virus; however, disease burden estimates due to other fever associated organisms, such as *Leptospira* and *Coxiella burnetii*, remain in question [9–13]. Recent research evaluating the status of global fever surveillance has identified significant gaps in the field, including the lack of multi-pathogen diagnostic approaches, as well as limitations in geographic and temporal coverage to account for disease variations observed across time and space [7,14–15].

Data regarding the cause of non-malarial febrile illness are currently lacking in Tanzania’s South-Central region. To identify the type and prevalence of various bloodstream and respiratory agents among febrile patients in Kilombero, Tanzania, we conducted a yearlong exploratory surveillance project, surveying the presence of 57 different microbial agents in blood and naso/oro-pharyngeal specimens. Additionally, we examined numerous correlates of febrile illness, and conducted demographic, clinical, and behavioral domain-specific multivariate regression to statistically establish associations with detection of identified agents.

## Methods

### Research ethics

Ethical clearance for this study was obtained from the National Health Research Ethics Sub-Committee (NatHREC) of Medical Research Coordinating Committee (MRCC) at the National Institute for Medical Research (NIMR/HQ/R.8a/Vol.IX/1735) in Tanzania, and the US Centers for Disease Control and Prevention, Center for Global Health (CGH HSR 2014–118). A written informed consent was obtained from each participant prior to the interview. Prior to enrollment, all patients provided written assent for participation. Pediatric patients (1 to 15 years-of-age) also needed documented parent consent in order to be enrolled. All pediatric patients were accompanied by an adult guardian at the time of interviewing. Guardians participated in assisting with completion of the pediatric questionnaires when necessary. For all adult participants who are unable to read or write, a study member read through the full document twice to ensure the patient had complete understanding of the terms and conditions associated with their enrollment in this study. If illiterate, the participant printed an “X” rather than their name on the informed consent form. This consent, as marked by an “X,” was then be verified with a signature of a witness. The participants were assured of anonymity in the analysis, presentation and publishing of the data.

### Study site

**Fig 1:**
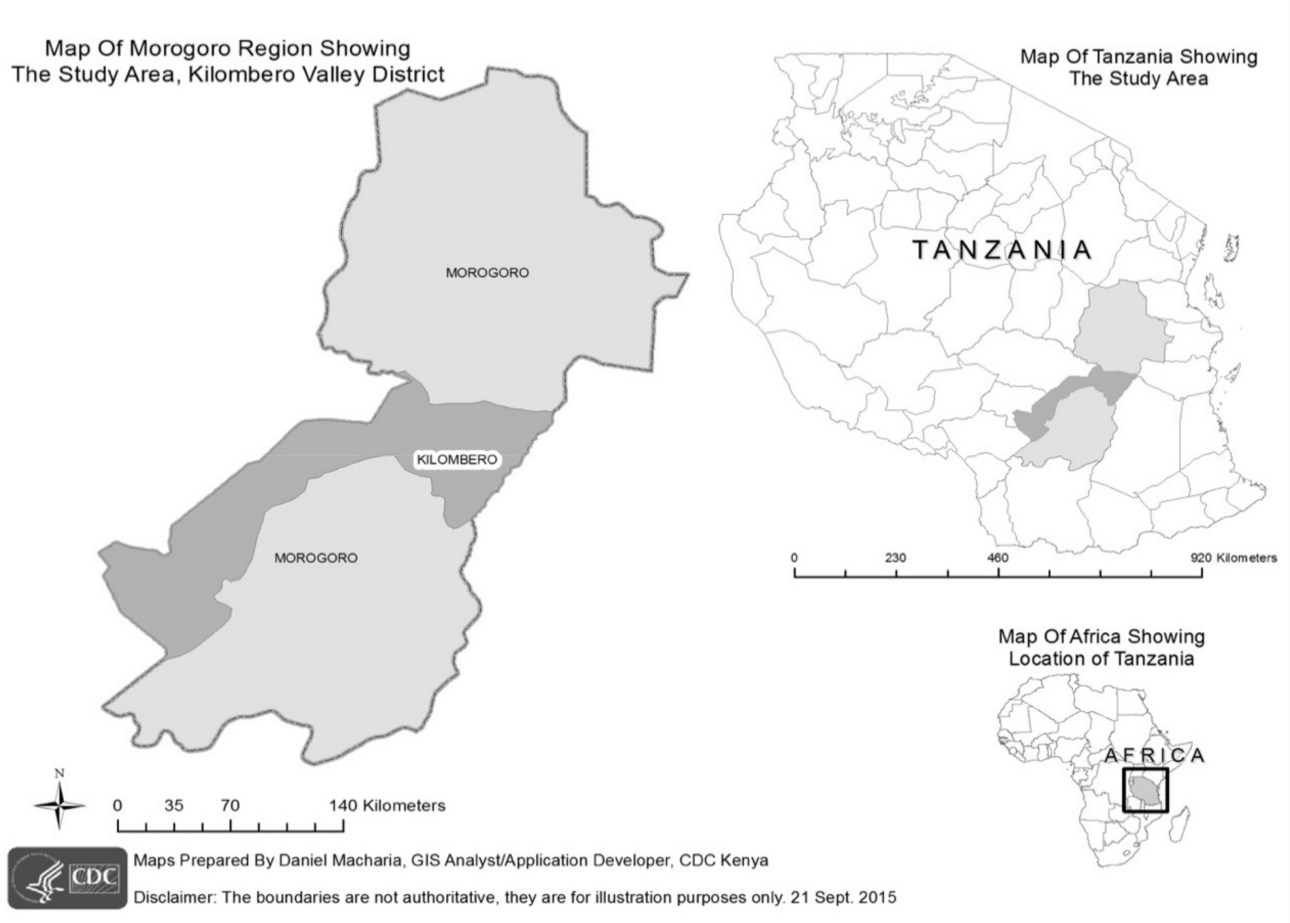
Map of study area in Kilombero district shown in dark gray in the Morogoro region inset.

This surveillance project was conducted in Tanzania’s Kilombero Valley, a rural area where agro-industrial land use associated with sugarcane production has situated a growing human population in close proximity to areas of increased wildlife biodiversity (Fig 1). The Illovo Sugar Limited Estate in Kilombero is the largest sugarcane production facility in Tanzania. The Estate is comprised of 13,000 hectares of land, of which 9,562 hectares are under cultivation. The Estate consists of nine farms and two factories, and is further surrounded by small family-owned farms. The Estate neighbors two national parks including: Mikumi National Park and Udzungwa Falls National Park.

This cross-sectional study was conducted at the Illovo Sugar Limited Estate Hospital (K1) and Clinic (K2). K1 Hospital is a private 80-bed hospital, serving Illovo employees and dependents as well community members from nine surrounding villages, including: Nyandeo, Msolwa Ujamaa, Mkula, Mangula, Kiberege, Msolwa Station, Ruaha, Kidogobasi and Luhembe. K2 Clinic is operational five days a week (Monday-Friday) and is only available to Illovo employees and dependents. Employees and dependents are offered medical care free-of-charge; other community members who are not affiliated with the Illovo Company are offered care however must pay a fee for service, which is partially subsidized by the Illovo Company.

Kilombero (population >321,000) is situated at an elevation of 300m above mean sea level, in the Morogoro Region (population >2 million) of South-Central Tanzania. The climate is characterized by a long rainy period (March-May) and a short rainy period (October-December). Malaria transmission intensity in this region is considered high, with 13% prevalence in community-based surveying of children (6–59 months) testing positive for malaria by rapid diagnostic testing [16–17].

### Study design

The objective of this hospital-based syndromic surveillance study was to examine exposure and epidemiologic associations with detection of bloodstream and respiratory agents among patients presenting to clinical settings with acute febrile illness (AFI) in Kilombero, Tanzania. Consenting patients, one year-of-age and above, with an axillary temperature of ≥37.5°C and symptomatic onset of ≤5 days, were eligible for enrollment. Blood and NP/OP specimens, along with demographic, clinical and behavioral data were collected in order to conduct epidemiologic analysis.

### Data collection

Patients at K1 Hospital and K2 Clinic were screened, and if found to be eligible for enrollment, they were directed to one of our local clinical staff members for processing. Clinical, epidemiologic and laboratory data were entered electronically onto Samsung tablets using an Open Data Kit (ODK) platform.

### Clinical assessment

Following screening and informed consent, a clinical officer or assistant medical officer conducted a detailed clinical history followed by a physical examination. The attending officer entered all clinical data in a standardized electronic clinical case report form on the tablets using ODK Collect. Data recorded on the eCRF included: patient vital signs, self-reported symptoms, self-reported previous and concurrent infections (i.e. malaria, HIV), provisional diagnoses, treatment recommendations, as well as final admission status.

### Epidemiologic evaluation

After the clinical examination and consultation was complete, clinical officers then administered a comprehensive epidemiologic survey to further capture demographic and behavioral data. Patients under 15 years-of-age were administered a pediatric epidemiologic survey, whereas patients 15 years-of-age and above were administered an adult epidemiologic survey. These two survey questionnaires were similar in scope, however tailored to include or omit certain age-specific inquiries (e.g., vaccination history for children, occupational activities for adults). Data recorded on the epidemiologic survey included: socio-economic data, household and residence status, travel history, wildlife and livestock animal interactions, recreational/occupational activities, as well as preventative measures (i.e. vaccinations, vector control measures, etc.). Wealth index was calculated using principal component analysis (PCA) so to describe socio-economic status (SES) differentiation within our study group. In this regard, we pilot tested several examples of household assets that could be appropriate for creating SES indices for this target population. The following six household assets were chosen for calculation of wealth index by PCA: radio, bicycle, mobile phone, light source, television, and refrigerator.

### Sample collection and storage

Prior to initiation of any antimicrobial therapy, blood was drawn from each enrolled participant. The venipuncture site was cleaned with an alcohol swab, followed by disinfection with povidone-iodine. For adults, a total of 15 mL of venous blood was extracted, including 10 mL of whole blood (in two 5mL EDTA tubes) for aliquotting and 5 mL of whole blood (in one 5mL non-EDTA plain tube) for further centrifugation of serum. For children, smaller volumes of 5–8 mL total whole blood were obtained. Blood samples were stored in a –80°C freezer on site, backed-up by a generator for emergency power supply.

Subsequently, at the time of enrollment, if any adult patient was suffering from respiratory conditions (cough, chest pain or sore throat) in addition to his/her febrile illness, staff collected an NP/OP swab. Given that pulmonary infections in children may present with non-specific signs, especially early in the infectious course, staff collected an NP/OP swab from all enrolled pediatric febrile patients irrespective of pulmonary symptoms. NP/OP swabs were placed in viral transport media (VTM) and stored in a –80°C freezer on site prior to specimen transport.

### Diagnostic investigations

An SD Bioline (pan/*pf*) malaria rapid diagnostic test (Standard Diagnostics, Inc.) was performed at point-of-care for all participants. If the patient yielded a positive rapid test result, a microscopic examination of Giemsa-stained thick blood film smears was performed to determine intensity of infection. Results were scored as 0 (no parasites), 1+ (10–19 parasites/μL), 2+ (20–29 parasites/μL), 3+ (30–39 parasites/μL), or 4+ (40 or more parasites/μL).

Whole blood and NP/OP specimens were transported by road, on dry-ice, to Kenya Medical Research Institute (KEMRI) laboratories in Nairobi, Kenya, on a quarterly basis, where they were stored in a –80°C freezer prior to undergoing diagnostic testing using the syndromic TaqMan Array Card (TAC) diagnostic platform [18–20]. For the purposes of this study, we utilized both acute febrile illness (AFI) and respiratory illness TAC assays to screen blood and NP/OP specimens respectively for a combined total of 57 viral, bacterial and parasitic organisms [18–19].

For whole blood processing, total nucleic acid was extracted from one aliquot (2.5 ml) of blood using High Pure Viral Nucleic Acid Large Volume Kit (Roche Diagnostics) and eluted in 100 μL of elution buffer. MS2 and PhHV were added to each sample to serve as built-in controls to confirm success of the extraction process and amplification efficiency for RNA and DNA targets [19]. We mixed 46 μL of total nucleic acid extract with AgPath One Step RT-PCR reagents (Life Technologies Corporation), in a 100 μL reaction, then pipetted into the inlet port of each channel. Cards were centrifuged (1 min, 1200 rpm twice), sealed and the inlet ports removed as directed by the manufacturer’s instructions. All AFI TACs were run on the ViiA™ 7 real-time PCR system (Life Technologies Corporation) using PCR cycling conditions comprising of 20 min at 45°C, 10 min at 95°C, followed by 45 two-step cycles of 15s at 95°C and 1 min at 60°C. Confirmed AFI TAC detection of a bloodstream agent was defined by a ≤37 threshold cycle (C_t_) value [19]. For 14 of the 27 agents detected by AFI TAC V2 including: Chikungunya, Crimean-Congo Hemorrhagic fever, dengue, Hepatitis E, Marburg, Rift Valley fever, Yellow fever, *Brucella*, *C*. *burnetii*, *Leptospira*, *Rickettsia*, *Salmonella enterica*, *Salmonella* serovar Typhi, and *Yersinia pestis*, duplicate ports were used to detect different conserved regions of the target organism. In the event of discordant results, individual real-time PCR (IRTP) was performed to determine final detection status. For additional quality control, IRTP was performed on 10% of all positives and 10% of all negatives.

For NP/OP processing, total nucleic acid was extracted from specimens in the KingFisher ML extraction platform (Thermo Scientific, Waltham, MA) using MagMax nucleic isolation kit (Life Technologies, Carlsbad, CA). Briefly, 100μl of NP/OP specimen was mixed with 260μl of lysis-binding solution and then washed once with 600μl wash solution I, and twice with 450μl wash solution II. Nucleic acid material was eluted in 60μl elution buffer. Forty-six microliters of total nucleic acid was mixed with AgPath One Step RT-PCR reagents (Life Technologies Corporation), in a 100 μL reaction, and pipetted into the port of each channel in the cards. The assays were run on the ViiA™ 7 real-time PCR system (Life Technologies Corporation) using PCR cycling conditions 45°C for 10 min, 94°C for 10 min, and 45 cycles of 94°C for 30 s followed by 60°C for 1 min [18]. Confirmed positive Respiratory TAC detection of a NP/OP agent was defined by ≤37 cycle threshold (C_t_) value. For all 30 agents detected by Respiratory TAC, duplicate ports were used to detect conserved regions of the target pathogen. In the event of discordant results, IRTP was performed to determine final detection status. Influenza A positives were further sub-typed by PCR to explore lineages based upon the hemagglutinin (HA) surface protein, that included InFA H1N5, InFA H3N2, InFA H1N1, InFA H5a and InFA H5b.

### Statistical analysis

Statistical analyses were performed using SAS version 9.3 software (SAS Inc., Cary, NC). Descriptive statistics were presented as medians, ranges, and interquartile ranges (IQR) for continuous variables and as proportions and charts for categorical variables. Given the high frequency of detection of *Plasmodium*, we further examined detection status across three diagnostic platforms, and conducted a non-parametric tests Kruskal-Wallis test to compare mean C_t_ values, as determined by qPCR, for patients grouped within each level of parasite intensity (1+, 2+, 3+, or 4+), as determined by blood smear. Additionally, we conducted a linear multivariate regression analysis to determine mean *Plasmodium* C_t_ values associated with examined independent variables.

Clinical and epidemiologic correlates of agents detected among at least 10% of our patient population were evaluated using an agent-specific filtered multivariate logistic regression approach to determine statistically significant factors among test-positive participants against a test-negative control group. Given the large number of potential risk factors examined in this study, we first assessed indicators by multivariate regression within respective domains, including socio-demographic, clinical and behavioral domains. Statistically significant factors, as determined by domain-specific regression, were then evaluated in a combined multivariate logistic regression model.

## Results

### Patient screening and enrollment

From 15 September 2014–13 September 2015, a total of 1104 AFI patients were screened to determine eligibility for enrollment, of which 1007 were officially enrolled and 997 completed an epidemiologic survey. The majority of enrolled participants (66%) were male. The median age of enrollment was 23 years (range 1–79 years).

Of the 1007 patients enrolled, 139 were children (1<5 yrs; 14%), 186 were older children (5<14 yrs; 19%) and 672 were adults (≥15 years-of-age; 67%). All enrolled patients were Tanzanian, 851 (85%) lived on the grounds of the Estate, and 823 (82%) were full-time residents of the Kilombero area. Among the enrolled adults, 249 (37%) were field laborers (e.g., sugarcane cutting and weeding) employed by the Estate. Educational levels varied among our adult population, with 479 (71%) patients having primary school-level education or below. Socio-economic status, as denoted by the wealth index, also varied greatly among all enrolled participants, with the majority of patients (57%) falling within the two poorest quintiles (Table 1).

**Table 1:** Demographic and socio-economic characteristics of enrolled febrile patients.

Indicator	Children: 1<5yrs (N = 139)	Older Children: 5<14yrs (N = 186)	Adults: ≥15 yrs (N = 672)	Total (N = 997)^†^
	n(%)	n(%)	n(%)	n(%)
**Gender**				
Female	68 (49)	90 (47)	176 (26)	33 4(33)
**Age (years)**				
Median (IQR)	2.6 (1.7–3.6)	9.2 (6.7–11.3)	31.2 (23.7–40.3)	23.8 (9.7–35.5)
Mean (SD)	2.7	9.3	32.8	24.2
**Residence**				
Live on Estate Grounds	110 (79)	159 (86)	582 (87)	851 (85)
Live Outside Estate Grounds	29 (21)	27 (15)	90 (13)	146 (15)
**Education Level**				
No formal education	N/A	69 (37)	11 (2)	216 (22)
Incomplete primary school	N/A	89 (48)	34 (5)	126 (13)
Completed primary school	N/A	20 (11)	434 (65)	454 (46)
Incomplete secondary school	N/A	8 (4)	57 (9)	65 (7)
Completed secondary school	N/A	N/A	112 (17)	112 (11)
Completed vocational school and/or university	N/A	N/A	22 (3)	22 (2)
**Residence Status**				
Full time	136 (99)	181 (97)	506 (75)	823 (83)
Part time	2 (1)	4 (2)	163 (24)	169 (17)
**Occupation at Illovo Estate**				
Sugarcane cutting	N/A	N/A	153 (23)	153 (15)
Weeding	N/A	N/A	96 (14)	96 (10)
Factory work	N/A	N/A	3 (1)	3 (1)
Managerial work	N/A	N/A	106 (16)	106 (11)
Security work	N/A	N/A	8 (1)	8 (1)
Other Estate work	N/A	N/A	92 (14)	92 (9)
Not employed by the Estate	N/A	N/A	214 (32)	214 (22)
**Wealth Quintile (based on household possessions)**				
1 (poorest)	13 (9)	17 (9)	187 (28)	217 (22)
2	53 (38)	61 (33)	237 (35)	351 (35)
3	6 (4)	5 (2)	19 (3)	30 (3)
4	55 (40)	84 (42)	209 (31)	348 (35)
5 (wealthiest)	12 (8)	19 (10)	19 (3)	50 (5)

**†** *Of the 1*,*007 participants*, *we captured 997 epidemiologic surveys*

The most common presenting complaints, other than fever, were headache (80%), cough (32%) and abdominal pain (19%) (Table 2). The mean axillary temperature was 38.4°C (range 37.5°C—40.5°C) which was consistent across all age groups. Given similarity in fever distributions among younger children, older children and adults, we could define fever tertiles of all enrolled febrile participants, defined as mild fever (37.5°C—38.0 °C), moderate fever (38.1°C—38.6°C), and severe fever (38.7°C—40.5°C). Each tertile comprised approximately 33% of all enrolled participants.

**Table 2:** Clinical characteristics of enrolled febrile pediatric and adult patients.

Indicator	Children (1<5yrs) (N = 139)	Older Children (5<14yrs) (N = 190)	Adult (≥15yrs) (N = 678)	Total (N = 1007)
	n(%)	n(%)	n(%)	n(%)
**Axillary Temperature (°C)**				
Mild (37.5°C—38.0°C)	87 (63)	117 (62)	377 (56)	581 (58)
Moderate (38.1–38.6°C)	45 (32)	59 (31)	233 (34)	337 (34)
Severe (38.7–40.5°C)	7 (5)	14 (7)	68 (10)	89 (9)
Median (IQR)	38.2 (37.8–38.7)	38.2 (37.9–38.7)	38.3 (37.9–39)	38.2 (37.8–38.9)
Mean (SD)	38.3	38.4	38.4	38.4
**Recent Weight loss**				
Yes	26 (19)	19 (10)	99 (15)	144 (14)
**Chief Complaints**				
Headache	47 (34)	154 (81)	601 (89)	802 (80)
Cough	72 (52)	64 (34)	189 (28)	325 (32)
Abdominal pain	18 (13)	43 (23)	131 (19)	192 (19)
Chest pain	4 (3)	19 (10)	147 (22)	170 (17)
Vomiting	28 (20)	37 (20)	95 (14)	160 (16)
Diarrhea	18 (13)	10 (5)	73 (11)	101 (10)
Sore throat	3 (2)	25 (13)	69 (10)	97 (10)
Other	19 (14)	33 (17)	189 (28)	241 (24)
**Presentation with Respiratory Illness**				
Yes	74 (53)	83 (44)	254 (38)	411(41)
**HIV Status (self-reported)**				
Positive	0 (0%)	1 (1)	20 (3)	21 (2)
Negative	8 (6)	9 (5)	194 (29)	211 (21)
Unknown	131 (94)	180 (95)	464 (68)	775 (77)
**Provisional Diagnosis**				
Malaria	24 (17)	73 (38)	295 (44)	392 (39)
Undefined Fever	30 (22)	40 (21)	120 (18)	190 (19)
UTI	26 (19)	31 (16)	118 (17)	175 (17)
Upper Respiratory Infection	33 (24)	43 (23)	88 (13)	164 (16)
Pneumonia	44 (32)	27 (14)	79 (12)	150 (15)
Lower Respiratory Infection	12 (9)	9 (5)	40 (6)	61 (6)
Tonsillitis	5 (4)	16 (8)	19 (3)	40 (4)
Typhoid	0 (0)	4 (2)	25 (4)	29 (3)
Sepsis	5 (4)	2 (1)	15 (2)	22 (2)
Bronchitis	1 (1)	4 (2)	15 (2)	20 (2)
Severe Anemia	5 (4)	2 (1)	2 (0)	9 (1)
**Admission Status**				
Admitted	48 (34)	68 (36)	347 (51)	463 (46)

HIV positivity, albeit self-reported, was low (2.1%). Of all enrolled participants, 463 (46%) were admitted for in-patient care and treatment (Table 2). The most common provisional diagnosis was malaria (39%), followed by “undefined fever” (19%), urinary tract infection (17%), and upper respiratory infection (16%). Patient-level data, inclusive of particular clinical and epidemiologic data as well as diagnostic results, are included in a supplemental file (S1 Table).

### Laboratory results

Malaria rapid testing was performed at point-of-care on 968 (96%) patients. Of these, 327 (34%) were positive for malaria, including 150 (16%) that were positive for *P*. *falciparum*, 6 (1%) that were positive for *Plasmodium* non-*falciparum* species, and 171 (18%) that were positive for both *P*. *falciparum* and non-*falciparum* species. Of the 327 malaria positives whereby a thick film blood smear was performed, 4 (1%) revealed no parasite found, 17 (5%) were denoted as 1+, 133 (41%) were denoted as 2+, 111 (34%) were denoted as 3+, and 62 (19%) were denoted as 4+.

Diagnostic evaluations using both AFI and respiratory TAC identified 31 different viral, bacterial and parasitic agents among 457 febrile patients contributing only blood specimens and 385 febrile patients contributing both blood and NP/OP specimens. There were 226 (25.6%) patients in which we did not detect nucleic acid for any of the 57 agents surveyed (Table 3). Multiple codetections were also observed using both TAC assays (Tables 4 and 5).

**Table 3:** Detections of viral, bacterial and parasitic agents by acute febrile illness (AFI) and respiratory TAC.

Agent Detected	Younger Children (1<5yrs)	Older Children (5<14yrs)	Adults (≥15yrs)	All ages
	n (%)	n (%)	n (%)	n (%)
**Blood Stream Agents**	**N = 58**	**N = 156**	**N = 628**	**N = 842**
*Plasmodium*	7 (12)	53 (34)	339 (54)	399 (47)
*Leptospira*	0 (0)	2 (1)	20 (3)	22 (3)
*Bartonella*	0 (0)	0 (0)	4 (1)	4 (1)
*Salmonella* non-Typhi	0 (0)	2 (1)	2 (1)	4 (1)
*Coxiella burnetii*	0 (0)	0 (0)	2 (1)	2 (1)
*Rickettsia*	0 (0)	0 (0)	2 (1)	2 (1)
West Nile virus	0 (0)	0 (0)	1 (1)	1 (1)
***Total number of bloodstream detections***	*7*	*57*	*370*	434
**Naso-Oro Pharyngeal (NP/OP) Agents**	** N = 85**	** N = 140**	**N = 160**	**N = 385**
*Haemophilus influenzae*	67 (79)	127 (91)	64 (38)	258 (67)
*Streptococcus pneumoniae*	59 (69)	93 (66)	61 (38)	213 (55)
*Moraxella cararrhalis*	57 (67)	68 (49)	27 (17)	152 (39)
*Staphylococcus aureus*	18 (21)	66 (7)	57 (36)	141 (37)
*Pseudomonas aeruginosa*	35 (41)	53 (38)	49 (31)	137 (36)
Human Rhinovirus	28 (33)	44 (31)	26 (16)	98 (25)
Influenza A	7 (8)	21 (15)	63 (39)	91 (24)
Enterovirus	25 (29)	25 (18)	7 (4)	57 (15)
*Klebsiella pneumoniae*	16 (19)	22 (16)	17 (11)	55 (14)
Group A *Streptococcus*	9 (11)	24 (18)	13 (8)	46 (12)
Adenovirus	14(16)	21 (15)	3 (2)	38 (9)
Respiratory Syncytial virus	6 (7)	3 (2)	3 (2)	12 (3)
Influenza B	0 (0)	4 (3)	7 (4)	11 (3)
Coronavirus 229E	4 (5)	6 (4)	1 (1)	11 (3)
Human metapneumovirus	4 (5)	0 (0)	5 (3)	9 (3)
Parainfluenza virus 1	5 (5)	2 (1)	1 (1)	8 (3)
Parainfluenza virus 3	6 (7)	0 (0)	0 (0)	6 (2)
Coronavirus OC43	0 (0)	3 (2)	2 (1)	5 (1)
*Mycoplasma pneumoniae*	1 (1)	3 (2)	0 (0)	4 (1)
Parainfluenza virus 2	0 (0)	2 (1)	1 (1)	3 (1)
*Bordetella pertussis* 1	1 (1)	0 (0)	1(1)	2 (1)
*Mycobacterium tuberculosis*	0 (0)	1 (1)	1 (1)	2 (1)
Parainfluenza virus 4	0 (0)	1 (1)	0 (0)	1 (1)
Coronavirus HKU1	0 (0)	1 (1)	0 (0)	1(1)
***Total number of NP/OP detections***	*362*	*590*	*409*	*1361*

**Table 4:** Frequency of detection of single and multiple organisms by the AFI TAC assay.

Detection Frequency	N	%
No organism detected	421	50.0%
One organism detected	414	49.2%
Codetection of 2 organisms	7	0.8%

**Table 5:** Frequency of detection of single and multiple organisms by the respiratory TAC assay.

Detection Frequency	N	%
No organism detected	10	2.6%
One organism detected	44	11.4%
Codetection of 2 organisms	71	18.4%
Codetection of 3 organisms	70	18.2%
Codetection of 4 organisms	78	20.3%
Codetection of 5 organisms	52	13.5%
Codetection of 6 organisms	40	10.4%
Codetection of 7 organisms	13	3.4%
Codetection of 8 organisms	7	1.8%

### Bloodstream detections

AFI TAC was performed on 842 febrile patients, of which we detected at least one bloodstream agent in 434 participants, including 7 (12%) of 58 younger children, 57 (37%) of 156 older children and 370 (59%) of 628 adults. Among these 842 surveyed participants, we detected nucleic acid for 7 different bloodstream agents, including: *Plasmodium* (399; 47%), *Leptospira* (22; 3%), *Bartonella* (4; 1%), *S*. *enterica* (4; 1%), *C*. *burnetii* (2; 1%), *Rickettsia* (2; 1%), and West Nile virus (1; 1%). Malaria was found to be the most common agent detected, with the prevalence of malaria increasing with age, as observed among 12% of younger children, 34% of older children and 54% of adults. All 20 cases of *Leptospira* were detected among adult patients.

### Naso/oro-pharyngeal detections

Additionally, respiratory TAC was performed on 385 febrile patients with cough, chest pain or sore throat, including 85 younger children, 140 older children and 160 adults. We detected nucleic acid for 24 different respiratory agents, including 12 viruses and 12 bacteria. The most prevalent agents detected include: *H*. *influenzae* (258; 67%), *S*. *pneumoniae* (213; 55%), *M*. *catarrhalis* (152; 39%), *S*. *aureus* (141; 37%), *P*. *aeruginosa* (137; 36%), Human Rhinovirus (98; 25%), influenza A (91; 24%), *K*. *pneumoniae* (55; 14%), Enterovirus (57; 15%), and group A *Streptococcus* (46; 12%).

Among bacterial organisms, *S*. *aureus* and *P*. *aeruginosa* demonstrated increasing prevalence among older age groups; whereas *M*. *catarrhalis* demonstrated decreasing prevalence with age. Among viral agents, we observed an increased prevalence of influenza among adult age groups, and a decreased prevalence of Adenovirus, Enterovirus and Respiratory Syncytial virus (RSV) with age.

### *Plasmodium* analysis

**Fig 2:**
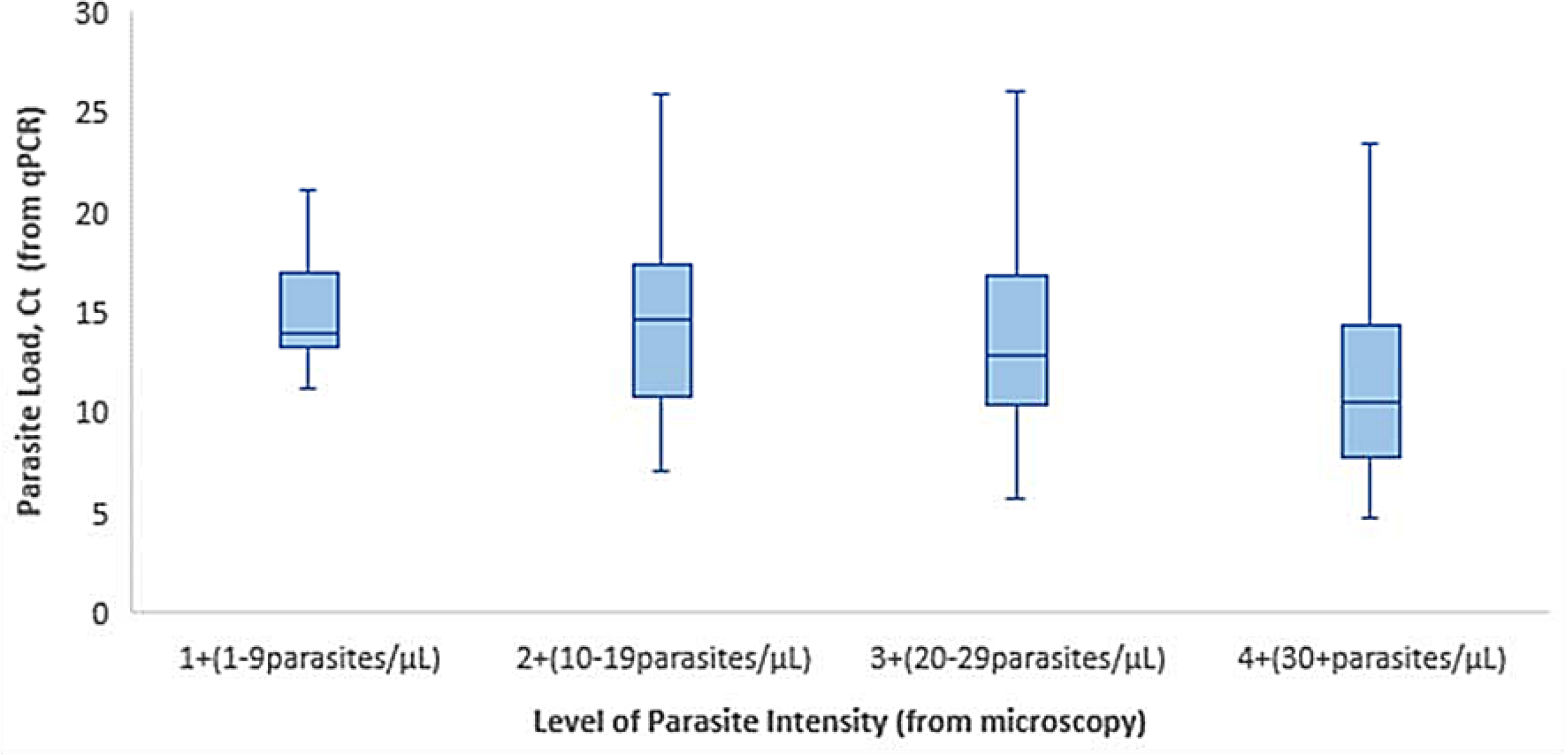
Box plots of parasite load (C_t_) by level of parasite intensity.

Cycle threshold (Ct) values were detected by using real-time quantitative PCR for all surveyed agents. Of the 399 *Plasmodium* positive cases, the average C_t_ was 17.9 with a range of 4.7–36.8. For the investigation of malaria, we used a tri-pronged diagnostic approach, including AFI TAC, an SD Bioline (pan*/pf*) malaria rapid diagnostic test and microscopy. To more thoroughly examine detection across these three diagnostic platforms, we conducted a non-parametric Kruskal-Wallis test comparing mean C_t_ values, as determined by qPCR, for patients grouped within each level of parasite intensity (1+, 2+, 3+, or 4+), as determined by blood smear. Since low C_t_ values correspond to high parasite loads, we would expect that lower mean C_t_ values correlate with higher levels of parasite intensity. Results were statistically significant (p-value = 0.0001) indicating differences among groups, and findings demonstrate an inverse relationship between mean C_t_ values and level of parasite intensity. As depicted in Fig 2, we observe lower mean C_t_ values (i.e. higher detected numbers of *Plasmodium* parasites), correlated with higher levels of parasite intensity.

**Fig 3:**
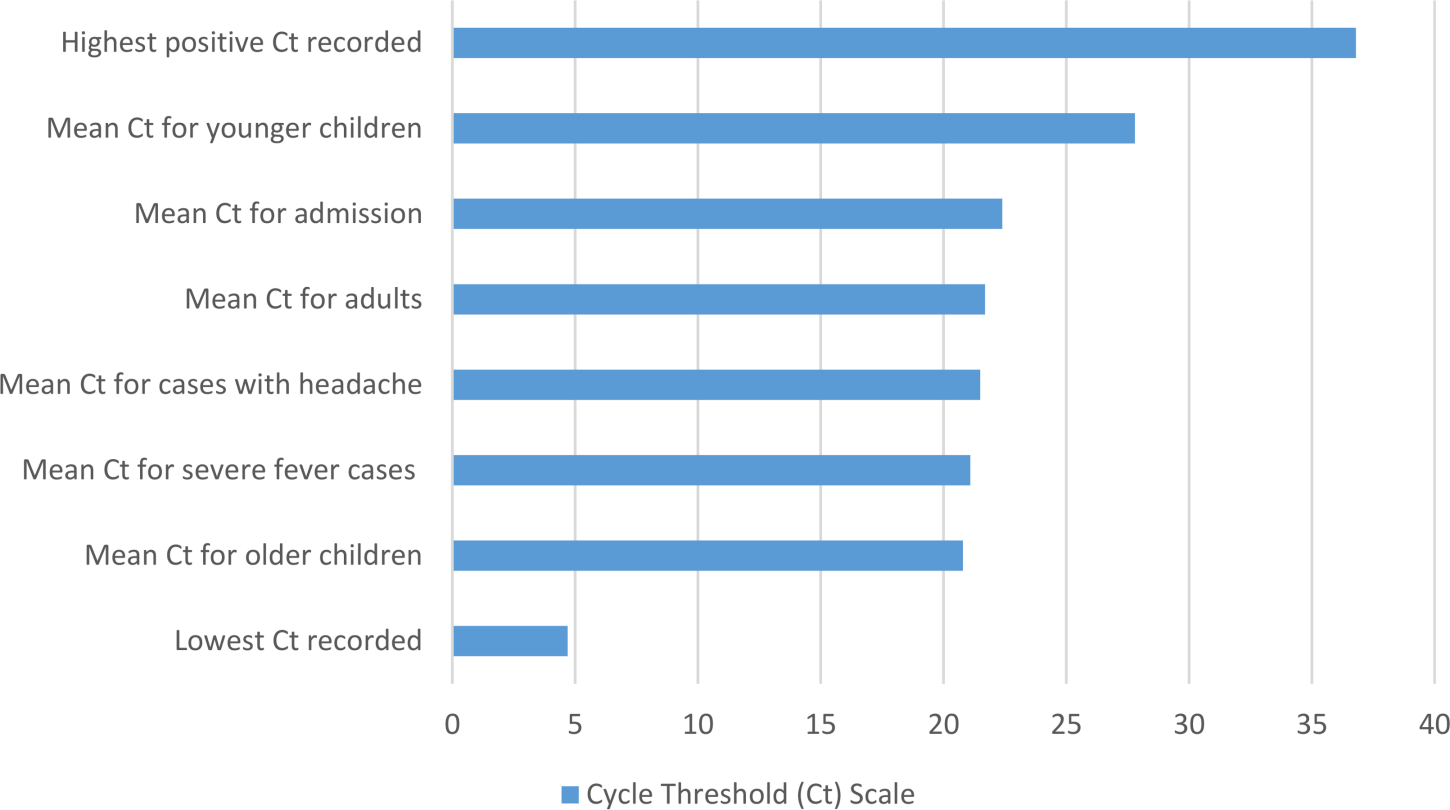
Cycle threshold (C_t_) scale for *Plasmodium* positive cases, denoting adjusted mean C_t_ values for demographic and clinical indicators.

Additionally, we conducted a linear multivariate regression analysis to determine if fully adjusted mean C_t_ values were associated with specific explanatory variables (Table 6). Outputs from this analysis demonstrate statistically significant lower mean C_t_ values for older children (20.8) as compared to younger children (27.8). In addition, we observe significantly lower mean C_t_ values for patients presenting with severe fever (21.1) and headache (21.5), as well as those who were admitted for in-patient care and treatment (22.4). Fig 3 denotes the statistically significant adjusted mean C_t_ values along a C_t_ scale of 1–37.

**Table 6:** Fully adjusted mean cycle threshold (C_t_) values associated with demographic and clinical factors as determined by multivariate linear regression. Dependent Variable: *Plasmodium* Cycle Threshold (C_t_); (Tested: 842; Positive: 399).

Variable	Categories	Adj. Mean C_t_	(Categories compared) p- values
Age group	(1) 15+yrs	21.7	(1–2) 0.477
	(2) 5-14yrs	20.8	(2–3) 0.036*
	(3) 1<5 yrs	27.8	(3–1) 0.058
Temperature	(1) Severe	21.1	(1–2) 0.002*
	(2) Moderate	24.5	(2–3) 0.882
	(3) Mild	24.7	(3–1) <0.001*
Respiratory Illness	Yes	25.5	< .0001*
	No	21.3	
Headache	Yes	21.5	0.006*
	No	25.4	
Abdominal Pain	Yes	24.3	0.096
	No	22.5	
Vomiting	Yes	22.7	0.193
	No	24.1	
Weight loss	Yes	23.9	0.379
	No	22.9	
Admission status	Yes	22.4	0.036*
	No	24.4	

*Statistically significant p-values

### Multivariate logistic regression

Clinical and epidemiologic correlates of agents detected by TAC among at least 10% of our patient population were evaluated using an agent-specific filtered multivariate logistic regression approach to determine statistically significant factors. Table 7 demonstrates the statistically significant findings from the final combined multivariate regression model.

**Table 7:** Statistically significant findings from domain-specific and combined multivariate regression model of prevalent agents detected.

Domain	Variables	Categories	Domain Adj. OR (95% CI)	P-value	Multivariate Adj. OR (95% CI)	P-value
**PLASMODIUM (Tested: 842; Positive: 399)**						
Socio-Demographic	Age	15+yrs	7.2 (3.2–16.3)	< .001*	5.7 (2.4–13.6)	< .001*
		5-14yrs	3.7 (1.6–8.7)	0.254	—	—
		1<5yrs	Ref	Ref	—	—
Clinical	Temperature	Severe	1.7 (1.2–2.4)	<0.001*	1.8 (1.3–2.7)	0.002*
		Moderate	1.0 (0.7–1.4)	0.107	—	—
		Mild	Ref	Ref	—	—
	Respiratory Illness	Yes	0.4 (0.3–0.6)	< .001*	0.4 (0.3–0.6)	<0.001*
		No	Ref	Ref	—	—
	Abdominal Pain	Yes	0.7 (0.5–0.9)	0.023*	0.7 (0.4–0.9)	0.03*
		No	Ref	Ref	—	—
	Admission status	Yes	2.1 (1.5–2.9)	< .001*	2.1 (1.5–2.9)	<0.001*
		No	Ref	Ref	—	—
**ENTEROVIRUS (Tested: 385; Positive: 57)**						
Socio-Demographic	Age	15+yrs	0.1 (0.0–0.2)	<0.001*	0.1 (0.03–0.2)	<0.001*
		5-14yrs	0.6 (0.3–1.2)	0.133	—	—
		1<5yrs	Ref	Ref	—	—
	Gender	Female	0.4 (0.2–0.9)	0.015*	0.5 (0.2–0.9)	0.02*
		Male	Ref	Ref	—	—
**INFLUENZA A (Tested: 385; Positive: 91)**						
Socio-Demographic	Age	15+yrs	9.7(3.9–24.2)	<0.001*	7.5 (1.3–24.4)	0.0004*
		5-14yrs	2.0 (0.8–5.4)	0.151	—	—
		1<5yrs	Ref	Ref	—	—
	Occupation	Estate employee- field laborer	0.4 (0.2–1.0)	0.037*	0.4 (0.2–0.9)	0.002*
		Estate employee- non-field laborer	1.4 (0.6–3.3)	0.386	—	—
		Non-Estate employee	Ref	Ref	—	—
Clinical	Cough (non productive)	Yes	6.0 (3.0–12.2)	< .0001*	6.7 (3.0–14.7)	<0.001*
		No	Ref	Ref	—	—
**GROUP A STREPTOCOCCUS (Tested: 385; Positive: 46)**						
Clinical	Sore throat	Yes	3.1 (1.4–6.6)	0.004*	3.6 (1.8–7.3)	<0.001*
		No	Ref	Ref	—	—
	Chest pain	Yes	0.2 (0.1–0.8)	0.017*	0.2 (0.1–0.5)	0.001*
		No	Ref	Ref	—	—
**HAEMOPHILUS INFLUENZAE (Tested: 385; Positive: 258)**						
Socio-Demographic	Age	15+yrs	0.2 (0.1–0.4)	< .001*	0.3 (0.1–0.6)	<0.001*
		5-14yrs	2.7 (1.3–5.9)	0.011*	2.8 (1.3–6.3)	<0.001*
		1<5yrs	Ref	Ref	—	—
	Residence Status	Yes	3.8 (1.0–14.4)	0.046*	0.1 (0.02–0.5)	0.006*
		No	Ref	Ref	—	—
**MORAXELLA CATARRHALIS (Tested: 385; Positive: 152)**						
Socio-Demographic	Age	15+yrs	0.1 (0.1–0.2)	< .001*	0.1 (0.05–0.2)	< .001*
		5-14yrs	0.5 (0.3–0.9)	0.028*	0.5 (0.3–0.9)	.04*
		1<5yrs	Ref	Ref	—	—
	Occupation	Estate employee- field laborer	8.5 (1.7–42.4)	<0.001*	7.4 (1.6–35.4)	0.007*
		Estate employee- non-field laborer	3.1 (0.6–16.4)	0.180	—	—
		Non-Estate employee	Ref	Ref	—	—
**PSEUDOMONAS AERUGINOSA (Tested: 385; Positive: 137)**						
Clinical	Cough (non productive)	Yes	0.4 (0.2–0.7)	0.009*	0.4 (0.3–0.9)	<0.001*
		No	Ref	Ref	—	—
	Chest pain	Yes	1.9 (1.1–3.3)	0.015*	1.9 (1.1–3.3)	0.016*
		No	Ref	Ref	—	—
**HUMAN RHINOVIRUS (Tested: 385; Positive: 98)**						
Socio-Demographic	Age	15+yrs	0.4 (0.2–0.8)	.006*	0.5 (0.2–0.9)	0.017*
		5-14yrs	0.9 (0.5–1.6)	0.720	—	—
		1<5yrs	Ref	Ref	—	—
**STAPHYLOCOCCUS AUREUS (Tested: 385; Positive: 141)**						
Socio-Demographic	Age	15+yrs	2.1 (1.1–3.9)	.0268*	2.1 (1.1–3.9)	0.548
		5-14yrs	3.3 (1.8–6.3)	<0.001*	3.3 (1.8–6.2)	<0.001*
		1<5yrs	Ref	Ref	—	—
**STREPTOCOCCUS PNEUMONIAE (Tested: 385; Positive: 213)**						
Socio-Demographic	Age	15+yrs	0.2 (0.1–0.4)	< .001*	0.3 (0.1–0.6)	<0.001*
		5-14yrs	2.7 (1.3–5.9)	0.011*	2.8 (1.3–6.3)	<0.001*
		1<5yrs	Ref	Ref	—	—
	Occupation	Estate employee- field laborer	2.8 (1.2–6.7)	0.021*	3.0 (1.3–7.1)	0.002*
		Estate employee- non-field laborer	1.0 (0.4–2.5)	0.947	—	—
		Non-Estate employee	Ref	Ref	—	—

*Statistically significant p-vlaues

## Discussion

This study provides an informative and valuable diagnostic and epidemiologic assessment of febrile illness in Kilombero, Tanzania among participants ≥ 1 year of age. Of 842 patients who contributed a blood specimen for diagnostic investigation, we were able to detect at least one microbial agent in 434 (52%) patients. Our study eligibility criteria and case definitions were not optimized to detect any specific agent. We maintained broad and inclusive enrollment criteria given the exploratory nature of our investigation.

Using our AFI TAC assay, we detected 7 different bloodstream agents among the 27 agents surveyed, including: *Plasmodium* (399; 47%), *Leptospira* (22; 3%), *Bartonella* (4; 1%), *S*. *enterica* (4; 1%), *C*. *burnetti* (2; 1%), *Rickettsia* (2; 1%), and West Nile virus (1; 1%). Other than one detection of West Nile virus, we did not detect any other surveyed arboviruses, including: dengue, chikungunya, Zika, Rift Valley fever and Crimean-Congo hemorrhagic fever. However, previous literature suggests that for the majority of arboviruses, viremia tends to peak prior to the onset of clinical illness, thus our acute sampling timeframe (0–5 days post symptom onset) might not have been optimized to detect such bloodstream agents [21–23].

The most prevalent agents detected by Respiratory TAC included: *H*. *influenzae* (258; 67%), *S*. *pneumoniae* (213; 55%), *M*. *catarrhalis* (152; 39%), *S*. *aureus* (141; 37%), *P*. *aeruginosa* (137; 36%), Human Rhinovirus (98; 25%), influenza A (91; 24%), *Klebsiella pneumoniae* (55; 14%), Enterovirus (57; 15%) and group A *Streptococcus* (46; 12%). While many of these agents are known to colonize in the human pharynx asymptomatically and thus not contribute to disease status, these organisms may have the capacity to launch an infectious course, especially in a host suffering from a weakened immune system and/or plagued by other concurrent infections. Previous studies have demonstrated that when influenza is identified, detection usually signifies etiology of clinical illness; thus, we are more confident in suggesting disease causality for the 101 (26%) individuals who presented with influenza A/B [24–26]. Among influenza cases, we detected influenza A in 91 (24%) patients, and detected influenza B in 11 (3%) patients. These results are similar to findings from a recent 30-month (2008–2010) nationwide sero-prevalence study of influenza in Tanzania, which found influenza A to be around eight times more prevalent than influenza B [27]; however, our results vary from analysis conducted the same year (2015–2016) in East Africa by the World Health Organization’s Global Influenza Programme, which detected similar prevalence of influenza A (55%) and influenza B (45%) [28].

In viewing the distribution of viral and bacterial respiratory agents across age groups ≥ 1 year of age, we observe increased frequency of detection of influenza A/B, *S*. *aureus* and *P*. *aeruginosa* with age, and a decreased frequency of detection of Adenovirus, Enterovirus, Respiratory Syncytial virus (RSV) and *M*. *catarrhalis*. A recent CDC TAC pilot study investigating the contribution of respiratory pathogens to hospitalizations of pneumonia in rural Western Kenya also utilized the respiratory TAC assay, among other diagnostic measures [29]. This research group found similar detection frequency of adenovirus (8%) and *S*. *pneumoniae* (60%); however, detected lower frequency of influenza A (8%) as compared to our study in Tanzania [29]. In another respiratory illness investigation by Jain *et al*. where TAC, PCR and serology were employed, researchers evaluated community-acquired pneumonia requiring hospitalization among US children [30]. While testing algorithms varied somewhat from the measures employed by this study, results from the US-based study demonstrated similar prevalence of influenza A (7%) and Human Rhinovirus (27%); lower prevalence of *S*. *aureus* (1%) and *S*. *pneumoniae* (4%); and higher prevalence of RSV (28%) and *M*. *pneumoniae* (8%), as compared to the younger pediatric cohort enrolled in our Tanzania-based study [30]. The variation observed in detection frequency across these distinct settings as well as others, suggests perhaps different existing exposures and/or epidemiologic risk depending upon geography [29–32]. Further multi-country TAC studies, using a standardized approach, with rigorous case definitions and uniform enrollment criteria, would help facilitate pooled analyses for more generalizable results.

Given that Kilombero is located in a highly endemic area for malaria, results are not generalizable and different diagnostic outcomes would be expected from non-endemic settings. Additionally, detection of *Plasmodium* in older children and adults is not unexpected, and positive detection is not necessarily indicative of fever etiology or resultant of new, active infection. To further examine quantification of *Plasmodium* detections across multiple diagnostic platforms, we conducted a non-parametric Kruskal-Wallis test comparing mean C_t_ values, as determined by qPCR, for patients grouped within each level of parasite intensity (1+, 2+, 3+, or 4+), as determined by blood smear. Findings from this analysis demonstrate an inverse relationship between mean C_t_ values and level of parasite intensity. Moreover, we conducted a linear multivariate regression analysis to determine fully adjusted mean C_t_ values associated with specific demographic and clinical variables. Outputs form this analysis show statistically significant lower mean C_t_ values for older children (20.8) as compared to younger children (27.8). Additionally, we observe lower mean C_t_ values for patients presenting with severe fever (21.1) and headache (21.5), as well as those patients who were admitted for in-patient care and treatment (22.4). As the clinical research community begins to shift away from traditional microscopy approaches and towards molecular diagnostics for the diagnosis of malaria, this type of analysis could be valuable for directing patient management and clinical decision-making based upon intensity of infection, as determined by quantitative C_t_ outputs [33].

While other investigational studies have relied upon laboratory outputs alone to characterize febrile participants, we supplemented our diagnostic findings with a robust epidemiologic analysis to further establish associations with detection status. To this end, clinical and epidemiologic correlates of agents detected among at least 10% of our patient population were evaluated using an agent-specific filtered multivariate logistic regression approach to determine statistically significant factors. Results demonstrated several statistically significant factors to be considered, particularly age and symptomatic presentation. Age was found to be statistically associated with detection of *H*. *influenzae*, *S*. *pneumoniae*, *M*. *catarrhalis*, *S*. *aureus*, Human Rhinovirus, Influenza A, Enterovirus, and *Plasmodium*. Symptomatic presentation of at least one clinical indicator examined was found to be statistically associated with detection of *Group A Streptococcus*, *P*. *aeruginosa*, Influenza A and *Plasmodium*. From this analysis, we also observe that detection of *Plasmodium* was most prevalent among participants presenting with severe fever (38.7°C—40.5°C) across all age groups. These results are in agreement with findings from another recent fever study by D’acremont *et al* which found malaria, along with pneumonia and typhoid, to be more common among patients presenting with severe illness as compared to those with mild illness in Tanzania [14].

Our study had several limitations, particularly with regard to limitations in our diagnostic approach. AFI-TAC analyzes whole blood specimens derived from venous blood, whereas respiratory TAC screens NP/OP swabs derived from a non-sterile pharyngeal source. While agents detected in whole blood indicate viremia, bacteremia, and/or parasitemia, agents detected from the pharynx may only be representative of microbial carriage. In this regard, for the purposes of this study, we did not state final determination of disease etiology. In order to keep consistent with our diagnostic definitions of positive detection, we only determined pathogen detection if quantitative PCR cycle thresholds (C_t_) were at or below 37. On certain occasions, particularly in the case of *S*. Typhi, patients presented with Ct values within just one point of our *a priori* determined C_t_ cutoff value (i.e., 37.04; 37.23; 37.99). It could be argued that C_t_ cutoffs need to be re-adjusted for certain pathogens included on the AFI TAC assay, particularly bacterial pathogens that may present with low copy numbers in blood. While uniform approaches to C_t_ cutoffs of syndromic TAC cards may enable diagnostic efficiency and ease-of-use for laboratory technicians, it may not account for the natural variations in microbial load for the diverse suite of viral, bacterial and parasitic agents surveyed by this assay. A better understanding of the diagnostic value of quantitative diagnostics in determining febrile disease etiology, particularly in situations of co-detection, as well as the utility of re-calibrating diagnostic thresholds (C_t_ cutoffs) for molecular tests, is critically needed [14]. Finally, while enrollment spanned one full calendar year to account for seasonal variation, we also note a limitation with temporal coverage, given the possibility of yearly variation. We suggest that syndromic surveillance projects of this kind be extended for a minimum of 3–5 years, in order to ascertain true prevalence estimates.

In light of these limitations, this study provides a novel platform for exploratory diagnostic and epidemiologic assessment of febrile illness in a previously un-surveyed region of sub-Saharan Africa. While previous studies have targeted specific epidemiologic variables and their relationship with diagnostic outcomes, this study surpassed normal epidemiologic investigative procedures, by exploring over 150 independent variables included in the clinical case report form (CRF) and epidemiologic survey. Given the large number of variables assessed in this study, the domain-specific and full multivariate approach to risk factor assessment provided a competent mechanism for data reduction. In this regard, our chosen statistical strategy allowed us to control for potential confounding factors, while adjusting for many factors that may demonstrate interaction.

Finally, a strong advantage of this study was our use of highly sensitive multi-pathogen molecular diagnostics. Quantitative diagnostics of this kind can not only expand the number of agents surveyed in our investigation, but can also assist in revealing the complexity of infectious disease diagnoses when multiple detections are observed. Previous studies suggest that co-infection of multiple microbial agents is likely to lead to pathogen interactions, which can have significant clinical ramifications [14]. The combination of multiple detections observed in this study underscores the difficulty in providing a simple diagnosis for febrile illness. However, as shown in the case of *Plasmodium*, C_t_ can add a useful quantitative metric for determining severity of infection and facilitate febrile patient processing, particularly in endemic settings when detection of parasitemia is not necessarily indicative of fever etiology. Given that the AFI TaqMan array card is optimized for AFI surveillance in the African region, it can serve as a rapid screen for outbreak investigation and/or for preliminary surveillance of circulating pathogens and is an appropriate diagnostic choice to fulfill the needs of our initial exploratory investigation. However, once baseline prevalence has been established, more target, pathogen-specific diagnostic approaches (such as RDTs) may be more cost-effective for directing public health surveillance and proper patient management.

## Conclusion

This study provides an informative and valuable diagnostic and epidemiologic assessment of febrile illness in Kilombero, Tanzania ≥ 1 year of age. The novelty of this study is proven in our use of highly sensitive multi-pathogen molecular diagnostics to better characterize the type and prevalence of agents detected among febrile patients which can lead to more rapid detection and control of diseases and enhanced global health security. Additionally, we provide an analysis of the association between adjusted mean C_t_ values for *Plasmodium* and key clinical and demographic variables, which may further inform clinical decision-making based upon intensity of infection, across endemic settings of sub-Saharan Africa.

## Supporting information

S1 Table Clinical, epidemiologic and diagnostic findings from enrolled participants. (XLS)
